# Metabolite Profiling in the Liver, Plasma and Milk of Dairy Cows Exposed to Tansy Ragwort (*Senecio jacobae*) Pyrrolizidine Alkaloids

**DOI:** 10.3390/toxins15100601

**Published:** 2023-10-06

**Authors:** Korinna Huber, Janine Saltzmann, Sven Daenicke

**Affiliations:** 1 Institute of Animal Science, University of Hohenheim, 70599 Stuttgart, Germany; 2 Institute of Animal Nutrition, Friedrich-Loeffler-Institute, 38116 Braunschweig, Germany; janine.saltzmann@fli.de (J.S.); sven.daenicke@fli.de (S.D.)

**Keywords:** pyrrolizidine alkaloid exposure, *Senecio jacobaea*, metabolite profiling, liver metabolism, dairy cow

## Abstract

Background: Plant-derived pyrrolizidine alkaloids (PAs) in feed cause metabolic disturbances in farm animals resulting in high economic losses worldwide. The molecular pathways affected by these PAs in cells and tissues are not yet fully understood. The objective of the study was to examine the dose-dependent effects of orally applied PAs derived from tansy ragwort in midlactation dairy cows. Methods: Twenty Holstein dairy cows were treated with target exposures of 0, 0.47, 0.95 and 1.91 mg of total PA/kg of body weight/d in control, PA1, PA2 and PA3, respectively, for 28 days. Liver tissue biopsy and plasma and milk samples were taken at day 28 of treatment to assess changes in metabolic pathways. A targeted metabolomics approach was performed to detect the metabolite profiles in all compartments. Results: The PA-affected metabolite profiling in liver tissue, plasma and milk revealed changes in three substrate classes: acylcarnitines (ACs), phosphatidylcholines (PCs) and sphingomyelins (SMs). In addition, in the plasma, amino acid concentrations were affected by PA exposure. Conclusions: PA exposure disturbed liver metabolism at many sites, especially devastating pathways related to energy metabolism and to amino acid utilization, most likely based on mitochondrial oxidative stress. The effects on the milk metabolite profile may have consequences for milk quality.

## 1. Introduction

Intoxication with tansy ragwort (*Senecio vulgaris*, Asteraceae) and other *Senecio* subspecies (spp.)-derived pyrrolizidine alkaloids (PAs) is commonly observed in plant-eating livestock [1] and is endemic in many regions of the world [2,3]. The ingestion of fresh plant material is low due to the unpalatability, but when alternative food is scarce, animals also graze on *Senecio* spp. in afflicted pasture areas. Furthermore, *Senecio* plants, when included in hay and silage, maintain the same PA contents and are then ingested voluntarily, because the taste of the alkaloids is no longer limiting the intake [2]. While acute toxicosis is rare, chronic toxicosis has a high economic impact due to animal losses. Up to 10% of cattle per year are intoxicated by PAs in South Africa [4]. PAs cause progressive liver damage and impaired liver function often without clinical symptoms until the final lethal stage [3]. The characteristic histopathology reveals hepatic megalocytosis, biliary hyperplasia, fibrosis and necrosis [5,6]. On a cellular level, the first site of action is the damage of the hepatic venous endothelium by oxidative stress and apoptosis, leading to a syndrome called hepatic sinusoidal obstructive syndrome [4]. PA hepatotoxicity is induced by downstream products of PA metabolism, the pyrroles. The hepatic cytochrome P 450 monooxygenases (CYP), which are located in mitochondria and microsomes, generate the reactive pyrrolic metabolites within the oxidative metabolism of xenobiotic and endogenous compounds [2]. This is called “metabolic activation” and is a prerequisite for PA-induced toxicities [7]. These reactive pyrrolic metabolites bind to DNA and proteins modifying the cellular metabolism, inducing apoptosis and thereby causing genotoxicity, cytotoxicity and hepatotoxicity [7]. In addition, as usual in oxidative pathways, reactive oxygen species (ROS) are generated most likely causing oxidative stress, and concomitantly, the hydroxylation of compounds could occur [4,7]. However, the molecular details of underlying pathological mechanisms lack scientific evidence and are not fully understood.

Only older scientific evidence about PA-induced hepatotoxicity in ruminants exists [3,5,6,8]; a detailed look at the hepatic metabolic processes as influenced by PA intoxication especially in dairy cows is missing. However, this issue of plant-related poisoning is still highly relevant for livestock production worldwide due to the huge economic losses [1]. More recent work about PA toxicity in dairy cows was performed confirming the carry-over of PA into the milk and thereby the transfer into the human nutritional chain [9,10]. Because an understanding of basic metabolic processes and potential toxic mechanisms is necessary to perform a risk assessment for the cow and also for the human milk consumers, the aim of this study was to examine central pathways of mitochondrial function, energy and substrate metabolism and biogenic amine production. Therefore, a targeted metabolomics approach using the Absolute IDQ p180 panel (Biocrates, Innsbruck, Austria) was performed to determine the metabolite profiles in the liver, plasma and milk in dairy cows treated with increasing dosages of PA applied intraruminally.

## 2. Results

**Figure 1:**
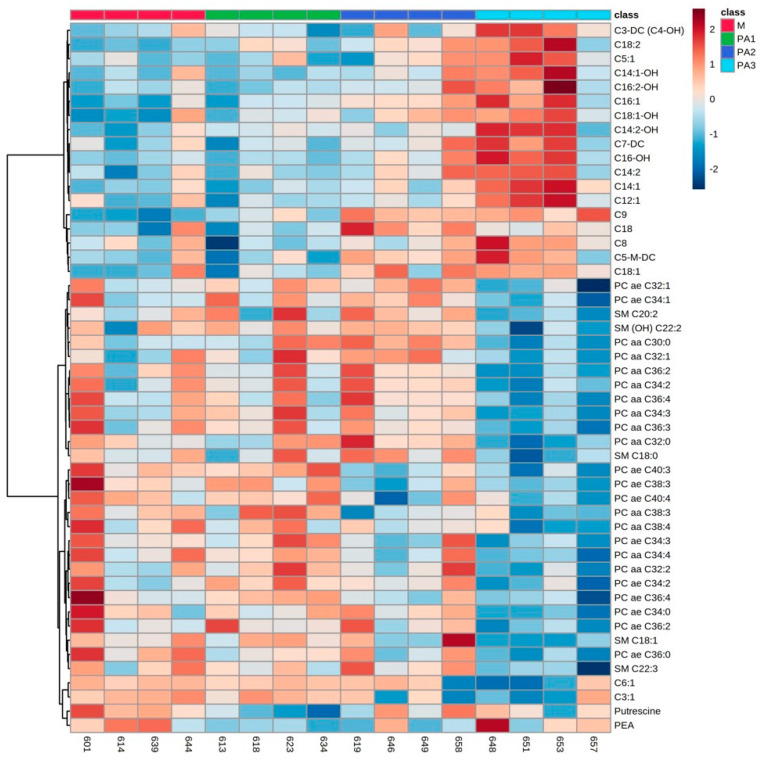
Heatmap of liver metabolite profiles of cows treated with different concentrations of pyrrolizidine alkaloids (M = Control, PA1 = 0.47 mg PA/kg body weight/day, PA2 = 0.95 mg PA/kg body weight/day and PA3 = 1.91 mg/kg body weight/day). Columns represent the metabolic profile of individual cows; rows demonstrate the top 50 most differentiating metabolites. Statistics for differences between treatment groups are demonstrated in Table 1.

**Figure 2:**
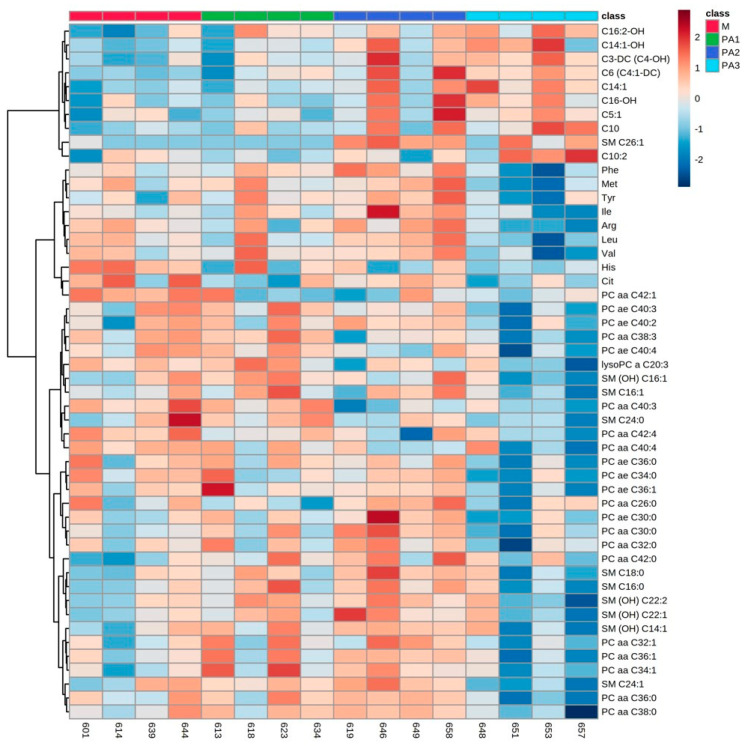
Heatmap of plasma metabolite profiles of cows treated with different concentrations of pyrrolizidine alkaloids (M = Control, PA1 = 0.47 mg PA/kg body weight/day, PA2 = 0.95 mg PA/kg body weight/day and PA3 = 1.91 mg/kg body weight/day). Columns represent the metabolic profile of individual cows; rows demonstrate the top 50 most differentiating metabolites. Statistics for differences between treatment groups are demonstrated in Table 2.

**Figure 3:**
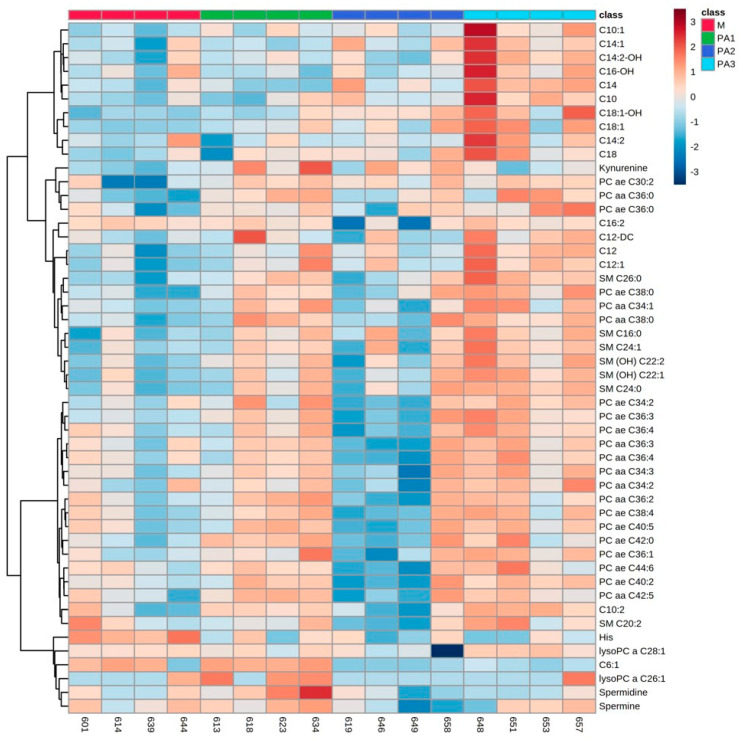
Heatmap of milk metabolite profiles of cows treated with different concentrations of pyrrolizidine alkaloids (M = Control, PA1 = 0.47 mg PA/kg body weight/day, PA2 = 0.95 mg PA/kg body weight/day and PA3 = 1.91 mg/kg body weight/day). Columns represent the metabolic profile of individual cows; rows demonstrate the top 50 most differentiating metabolites. Statistics for differences between treatment groups are demonstrated in Table 3.

As a major observation, PA-affected metabolite profiling in all compartments, the liver tissue, plasma and milk, revealed major changes in three substrate classes: acylcarnitines (ACs), phosphatidylcholines (PCs) and sphingomyelins (SMs). In addition, in plasma, amino acid concentrations were affected by PA exposure. Individual animals and their metabolite profiles were analyzed by heatmaps and are demonstrated in Figure 1 (liver), Figure 2 (plasma) and Figure 3 (milk). The total variation between treatment groups was visualized by partial least square-discriminant analyses (PLS-DA) for the liver (Supplementary Figure S1), for the plasma (Supplementary Figure S2) and for the milk (Supplementary Figure S3) including their Q2 values. 

### 2.1. Liver Metabolite Profiles

The top 50 hepatic metabolites of interest belonged mainly to AC, PC and SM. In general, short-, medium- and long-chain AC concentrations were elevated with increasing dosages of PA, while PC and SM concentrations were decreased. Many of the ACs also expressed higher concentrations of their hydroxylated forms (C3-DC (C4-OH), C14:1-OH, C16:2-OH, C18:1-OH, C14:2-OH and C16-OH). Furthermore, a high interindividual variation was observed in single animals per exposure group, which responded differently compared to the other members of the respective group (especially cow numbers 657, 658 and 646). Due to that interindividual variation, statistical significance between groups was less pronounced; however, most of the ACs expressed significantly higher concentrations with the highest PA dosage in group PA3 (Table 1). 

**Figure 4:**
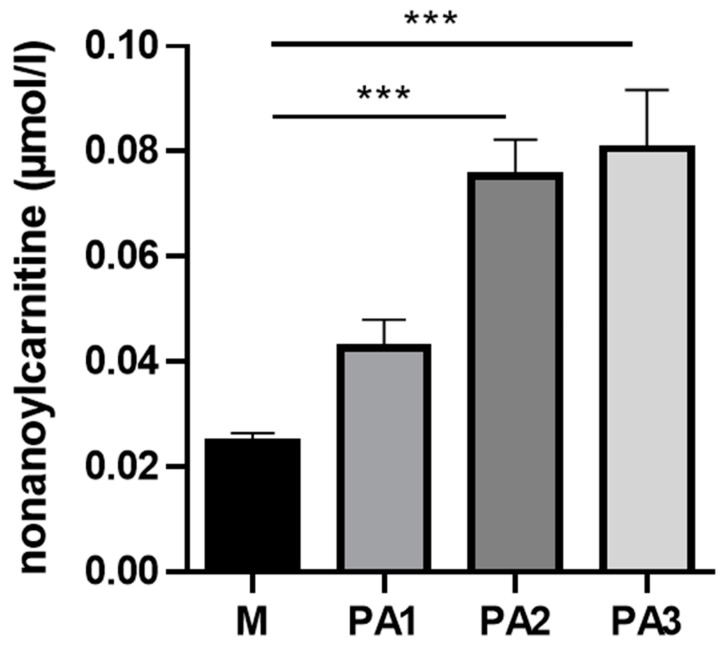
Liver nonanoylcarnitine (C9) concentrations in µmol/L of cows treated with different concentrations of pyrrolizidine alkaloids (M = Control, PA1 = 0.47 mg PA/kg body weight/day, PA2 = 0.95 mg PA/kg body weight/day and PA3 = 1.91 mg/kg body weight/day). Bars reflect means +/− SEM, *n*= 4 animals/treatment group. Statistical analysis was performed by using One-Way ANOVA with Dunnett´s multiple comparison test M versus PA 1, 2 and 3; effect of treatment was significant with *p* < 0.001. Asterisks indicate significance level of the post-test with *** *p* < 0.001.

Within the class of ACs, nonanoylcarnitine (C9) was detected with the strongest significant differences, showing higher concentrations in PA2 and PA3 compared to the control group M (Figure 4). Furthermore, the interindividual variation was low for C9; all animals of the two groups had equally high concentrations.

According to the heatmap, PC and SM expressed the lowest concentrations in the PA3 group; however, the huge interindividual variation and the low number of animals per group led to only weak statistical significances. Table 1 demonstrates exemplarily the statistical relevance of specific PCs (PC ae C32:1) and SMs (SM C20:2). Among biogenic amines, the concentration of putrescine was significantly lower in the PA1 group, and the concentration of phenylethylamine (PEA) did not express any significant differences compared to control 2 group M (Table 1).

### 2.2. Plasma Metabolite Profiles

In plasma, treatment with PAs mainly affected the concentrations of several ACs, amino acids (AAs), PCs and SMs, with enhanced concentrations of ACs and decreased concentrations of AAs, PCs and SMs compared to control 2. The results for ACs, AAs and exemplarily for PCs (PC aa C42:1 and PC ae C40:3) and SMs (SM C26:1 and SM C16:1) are demonstrated in Table 2. Many of the ACs occurred in their hydroxylated form (C16:2-OH. C14:1-OH, C3-DC (C4-OH) and C16-OH). Again, a huge interindividual variation was observed between cow responses to PA exposure. Cow numbers 658 and 646 are again quite different in their metabolite profile changes due to the increased application of PAs (Figure 2). 

### 2.3. Milk Metabolite Profiles

In milk, exposure to PAs mainly affected the concentrations of several ACs, PCs and SMs, with enhanced concentrations due to PAs. The results for ACs and exemplarily, for PCs (PC ae C32:1) and SMs (SM C26:0) are demonstrated in Table 3. Three ACs occurred in their hydroxylated form (C14:2-OH, C16-OH and C18:1-OH). One short-chain AC, C6:1, was significantly reduced in PA2 and PA3. Concentrations of kynurenine, histidine (His), spermidine and spermine are also affected by PA treatment (Table 3). Interindividual variation is less prominent; however, cow number 658 again deviated in its response to the milk metabolite profile from the responses of other PA2 group members (Figure 3).

## 3. Discussion

This study aimed to detect the influence of orally applied PAs on the metabolic pathways reflected by the metabolite profiles in the liver, plasma and milk of midlactating Holstein dairy cows. Oral treatment with PA toxins is technically demanding, and due to animal welfare issues, the number of animals per group in this study was kept small. However, the in-depth analysis of different body compartments by using metabolite profiling enabled us to define metabolic responses to this PA treatment in dairy cows. Keeping this experimental restriction in mind, the following discussion tried to interpret those findings critically and with care. Furthermore, this approach can provide ideas for future research in the field of alkaloid toxins and their biological impact. The success of the oral treatment was confirmed by dose-dependent increases in the plasma PA concentration with 22.6 ± 3.2 ng/mL in PA1, 69.2 ± 0.83 ng/mL in PA2 and 116.2 ± 8.5 ng/mL in PA3 [11].

### 3.1. Acylcarnitines in Liver, Plasma and Milk

Acylcarnitines (ACs) and carnitine homeostasis play an important role in the energy metabolism of an organism. Short-chain (C3–C5), medium-chain (C6–12) and long-chain ACs (C14–20) exist in plasma, most likely derived from tissue sources, especially from liver mitochondria and peroxisomes [12]. Scientific evidence reveals that ACs are biomarkers of impairment of fatty acid and branched-chain amino acid oxidation and signal mitochondrial dysfunctions. In the hydroxylated form, ACs represent oxidative stress [13]. However, ACs, especially medium- and long-chain ACs are also found to be physiological signals switching liver metabolism to produce ACs for use in brown adipose tissue for thermogenesis during cold exposure [14]. Furthermore, AC homeostasis also regulates free coenzyme A availability in tissues [13]. Thus, changes in plasma AC are difficult to interpret in regards to their impact. In this study, exposure of midlactating dairy cows to PA implemented a cascade of events reflected in the liver, plasma and milk and affected AC homeostasis, thereby demonstrating a causal relationship between PA toxins and metabolic consequences throughout the body. PA toxins damaged mitochondrial and microsomal Cyp 450 enzymes, increased intracellular oxidative stress and maybe impaired other nonidentified cellular processes [7]. Interestingly, changes in the AC of dairy cows were compartment-specific. While in liver tissue, hydroxybuturylcarnitine (C3-DC (C4-OH)), nonanoylcarnitine (C9), tetradecenoylcarnitine (C14:1) and hydroxydecanoylcarnitine (C16-OH) were the most enhanced by the highest dosage of PA (PA3), in plasma, only tetradecenoylcarnitine (C14:1) and in milk only tetradecanoylcarnitine (C14) concentrations were mainly enhanced. Hexenoylcarnitine (C6:1) was strongly diminished in milk. C14:1 in plasma might be released by the liver, which is discussed to be the major source of plasma AC [15]. Because concentrations in plasma are the sum of the influx and efflux of AC from the hepatic compartment, variations in the AC profile could be expected; thus, the plasma ACs inadequately reflect tissue acylcarnitine metabolism [16]. In milk, the activity of the mammary gland epithelial cells most likely modulates the AC profile independently. The liver as the first PA contact tissue expressed a high proportion of short-, medium and long-chain hydroxylated ACs reflecting the oxidative stress with consequences for the cellular metabolism. In addition, the high number of affected medium- and long-chain ACs pointed to a disturbed mitochondrial utilization of fatty acids resulting in this accumulation of ACs. This was protecting the hepatocytes against acyl-CoA accumulation and provided the possibility to release high-energy substrates to the periphery. 

One AC in liver was strongly higher expressed with PA2 and PA3 dosage, the odd-chain C9. Degradation of odd-chain fatty acids reveals acetyl-CoA and propionyl-CoA; the latter is converted to succinyl-CoA as an anaplerotic substrate for the citric acid cycle. This pathway was detected to restore energy production in myopathies mediated by long-chain fatty acid oxidation disorders [17]. However, PA intoxication appeared to impair especially odd-chain fatty acid utilization, but it is unclear which step is disturbed in this break-down process. Damage to mitochondrial respiration by PA intoxication most likely led to a feedback inhibition of pathways involved in the production of reducing agents, such as NADH, e.g., beta-oxidation and citric cycle [18]. The break-down of even-chain fatty acid provides acetyl-CoA only, which can be converted to ketone bodies and released into the blood, no longer loading the citric cycle. Furthermore, the beta-oxidation flux was significantly reduced leading to the accumulation of medium- and long-chain ACs in the liver. The pronounced break-down of odd-chain fatty acids would fuel the citric acid cycle twice, by acetyl-CoA and succinyl-CoA; thus, this pathway was blocked, maybe as an adaptive response to the decrease in respiratory chain activity, and thus there was less capacity to utilize reducing agents for ATP synthesis due to PA exposure. Therefore, C9 accumulated in liver tissue, but not in plasma. 

In milk, only even-chain ACs (medium and long-chain) accumulated with PA intoxication, also indicating disturbed beta-oxidation and the utilization of fatty acids in the mitochondrial respiratory chain. However, one exception was observed in the C6:1 concentration. This short-chain AC decreased strongly with PA2 and PA3 dosages. Assuming that the milk AC concentrations reflect mammary gland epithelial cell AC concentrations and assuming that lower C6:1 reflects lower hexenoic acid concentrations in epithelial cells, this decrease in C6:1 might reflect an adaptive process to improve mitochondrial energy production despite the damage by PA toxins in an organ-specific manner. Hexenoic acid is described to inhibit pyruvate oxidation at higher concentrations [19]. Therefore, a reduction stimulated pyruvate utilization instead of fatty acid utilization as a response to disturbed mammary gland epithelial cell metabolism of dairy cows exposed to PA.

### 3.2. Amino Acids in Plasma and Milk

Concentrations of Phe, Met, Ile, Arg, Leu and Val were significantly reduced in plasma with the highest dosage of PA (PA3). In milk, only His was strongly reduced by all three dosages of PA (PA1–3). Metabolic activation of PA reveals reactive pyrrolic metabolites, which were shown to bind to proteins and amino acids [7]. As an example, valine could be bound by dihydropyrrolizine alkaloids (DHP) as primary pyrrolic metabolite DHP-valine [20]. In principle, these adducts develop cytotoxic effects. However, pyrrole–amino acid adducts were also eliminated by urine, as shown for pyrrole-7-cysteine, pyrrole-9-cysteine, pyrrole-9-histidine and pyrrole-7-acetylcysteine in PA-intoxicated rats and humans [21]. Thus, reduced amino acid concentrations in the plasma of the dairy cows might reflect an increased use in building pyrrole–amino acid adducts to eliminate the toxic load via the kidneys. However, because the decrease was only observed with the highest PA dosage, it could also reflect a decrease in hepatic capacity to release amino acids into the plasma or an increase in uptake of amino acids in the peripheral tissues to use them as an alternative energy substrate due to disturbed beta-oxidation. In milk, only histidine was significantly reduced dose-dependently at any of the given PA dosages. Pyrrole-9-histidine was excreted by urine in human patients for several months after exposure to PA [21]. Lower histidine concentrations in the milk of cows treated with PA suggested a higher concentration of pyrrole-9-histidine thereby reflecting an excretion pathway of pyrrole–amino acid adducts via milk. Furthermore, renal excretion of pyrrole-9-histidine might limit availability of histidine for metabolism of mammary gland epithelial cells. However, detailed mechanisms about PA degradation and excretion are largely unknown and need further investigation. Histidine limits milk protein synthesis in dairy cows [22,23]. Thus, with reduced availability of histidine as indicated by lower milk histidine concentrations, less milk protein could be synthesized. 

Derivatives of amino acids, biogenic amines, were transiently affected by PA intoxication. In general, polyamines do have an important role in cellular growth and differentiation. In the liver of PA-exposed cows, putrescine was the lowest at the PA1 dosage indicating liver cell metabolic stress. Putrescine is known to be necessary for liver cell regeneration after ethanol intoxication [24]. At higher PA dosages, putrescine synthesis or uptake into the liver cells might be stimulated as an adaptation to increasing PA load and mitochondria damage. The pyrrolizidine alkaloid monocrotaline was stimulating ornithine decarboxylase activity (OCD), which is the key enzyme for putrescine, spermine and spermidine synthesis [25]. Analogously, PA might also stimulate OCD activity in the livers of PA-intoxicated cows to maintain putrescine levels. In milk, spermine and spermidine, downstream metabolites of putrescine degradation, were fluctuating in their concentrations, most likely reflecting metabolic stress in mammary gland epithelial cells, too.

### 3.3. Glycerophospholipids and Sphingolipids in Liver, Plasma and Milk

The interpretation of the findings in regards to specific complex lipids in liver, plasma and milk are difficult to interpret, because the biological meaning of these lipids is not yet well described. The strong increase in sphingomyelin SM C26:0 in milk at the PA3 dosage might reflect that the mammary gland epithelial cells initiated an adaptive response of anti-inflammation to protect the epithelial cells. Sphingomyelins expressed protective properties on human gut epithelial cells when ingested with cow’s milk [26]. Thus, this adaptive response of mammary epithelial cells might have somehow positive consequences for the consumers of cow milk exposed to PA.

### 3.4. Individual Responses to PA

As described before, the interindividual variation of metabolic response to PA exposure was obvious, especially in the liver metabolite profiles. Cows 657 (PA3), 658 (PA2) and 646 (PA2) expressed clear differences as visualized by the heatmap (Figure 1). A key response to PA3 exposure was the increase in all ACs; all animals of this group expressed this response, but cow 657 did not. This cow had the highest AST (323 U/L–mean of control M: 123.5 U/L) and GLDH (308 U/L–mean of control M: 32 U/L) activities in plasma and showed the strongest histopathological changes in the liver reflecting strong liver damage due to PA exposure (liver enzyme and histology data [11]). Liver cells were no longer able to synthesize AC at this stage of individual liver damage as indicated by the low concentrations of all ACs (with the exception of C9 (Table 1). Cows 658 and 646 showed higher AC concentrations compared to the two other cows of the PA2 group, indicating a more sensitive response to PA. Thus, PA-based mitochondria dysfunction revealed an adaptive response with higher AC synthesis at lower concentrations of PA. This higher individual sensitivity to PA was also reflected by the activity of liver enzymes in plasma. While AST (cow 658–195 U/L; cow 646–142 U/L–mean of control M: 123.5 U/L) was only slightly affected, the GLDH activity was strongly enhanced (cow 658–205 U/L; cow 646–153 U/L–mean of control M: 32 U/L) [11]. The other two cows of the PA2 group expressed low GLDH activity in plasma with 9 and 19 U/L. In the plasma, especially cows 658 and 646 of group PA2 showed high concentrations of AC (Figure 2) reflecting the higher synthesis of AC in liver cells combined with the capacity to release the molecules into the blood. In milk, variation between animals was again prominent in group PA2 with cow 658 (Figure 3). However, this variation was not reflected in AC, but mainly in phosphatidylcholine (PC) and sphingomyelin (SM) concentrations, which were higher than in the other animals of group PA2.

## 4. Conclusions

Although this study consisted of only four cows per treatment group, the targeted metabolomics approach revealed deep insights into the pathways affected by PA. Furthermore, comparing three different body compartments (liver, plasma and milk) at the same time, the dynamics of PA effects throughout the body were detected with clear compartment-specific responses. Finally, high variation between cow responses demonstrated the strong individuality and sensitivity of cows toward PA exposure. The findings suggest more research is needed to identify the exact underlying mechanisms of PA effects and to find the key targets mediating PA effects in the metabolic network. Furthermore, identifying the reasons for the variation in sensitivity toward PA in individual cows might enable the breeding of dairy cows that are more protected against chronic PA intoxication.

## 5. Materials and Methods

Within the main experiment, which is described in detail elsewhere [11], feed intake, oral and inner PA exposure, animal performance, clinical biochemistry and health variables were assessed. The trial was conducted at the experimental station of the Friedrich-Loeffler-Institut (FLI), Braunschweig, Germany, in agreement with the German Animal Welfare Act accepted by the Lower Saxony State Office for Consumer Protection and Food Safety (LAVES), Germany (file number 33.19-42502-04-19/3191_approval date 30 August 2019). In order to standardize the PA exposure levels to BW, an extract from dried tansy ragwort (*Senecio vulgaris*) was used and administered once daily in a chronic exposure scenario for 28 days. In short, the used tansy ragwort (*Jacobaea vulgaris* Gaertn.) was harvested in the summer of 2019 from a meadow in northern Germany, which was naturally covered. The tansy ragwort was thereby picked by hand without the root. After drying the plant material, the PA extract was prepared using aqueous methanol (90%) (Roth, Karlsruhe, Germany) in several extraction steps. Afterward, the methanol was removed via evaporation (Phytoplan, Heidelberg, Germany). The total concentration of PA in the extract was 4314.2 (analysis performed by the German Federal Institute for Risk Assessment) and 4184.4 (analysis performed by the Federal Research Institute for Animal Health, FLI) mg/kg. Compositions of PA alkaloids are described in detail elsewhere [11]. The amount of total carbohydrates in the PA extract was 342.0 g/kg, mainly consisting of glucose (38.5 g/kg), sucrose (133.5 g/kg) and fructans (57.5 g/kg) [11]. The amount of applied extract was calculated based on the PA concentration of the extract and the mean body weight (BW) prior to the beginning of the experiment and was targeted to reveal exposures of 0.47, 0.95 and 1.91 mg total PA/kg body weight/d in groups PA1, PA2 and PA3, respectively. The calculation of the target PA exposure for cows was determined on the basis of the literature data.

Because the PA extract contained unexpectedly high amounts of sugars, two control groups were tested. While group control 2 (molasses) received similar volumes of molasses as compared to the total extract amount of group PA3, the group control 1 (water) was administered a similar volume of tap water to test the additional hypothesis that the sugar present in the PA extract would not exert an extra effect on the investigated endpoints. However, as clinical–chemical traits did not suggest an extra effect of sugars present in the PA extract [11], only group control 2 (molasses, CON) was tested in the present investigation.

In the complete experiment, 20 pluriparous, nonpregnant, clinically inconspicuous lactating German Holstein cows (169.7 ± 30 days in lactation; mean ± standard deviation (SD)) were randomly assigned to the 5 treatment groups (*n* = 4 per group) considering comparable milk yield (39.7 ± 6.4 kg/day) and body weight (649 ± 51 kg). All cows received the same TMR, which consisted of maize silage (30%), grass silage (30%) and concentrate feed (40%) on a dry matter (DM) basis for ad libitum consumption. PA extracts and molasses were administered daily after the morning milking using an ororuminal tube described by [27]. 

All samples were taken at day 28 of the experiment. A volume of 2 mL milk was taken during the morning milking into cryotubes. Blood samples were collected 90 min after PA administration from an external jugular vein into EDTA-containing tubes. Blood samples were centrifuged at 2000× *g* for 15 min at 15 °C to harvest plasma. Liver tissue samples of approximately 200 mg were immediately collected after slaughtering from the isolated liver by using an automated spring-loaded biopsy instrument (Bard Magnum, Bard, UK). The location chosen for biopsy collection was consistently the *Lobus dexter hepatis, Facies parietalis*, closest to the location of liver biopsy sampling in in vivo. Liver and milk samples were shock-frozen in liquid nitrogen. All samples were stored at −80 °C until metabolite profiling.

The metabolite profiles in hepatic, plasma and milk samples of 16 cows (*n* = 4 per group, CON, PA1, PA2 and PA3) were determined as reported previously [28,29]. For tissues, the preparation protocol was slightly adapted. In short, liver tissue samples (around 100 mg) were homogenized in ethanol/phosphate buffer (85:15) (Roth, Karlruhe, Germany/Sigma-Aldrich Chemie GmbH, Taufkirchen, Germany) by mechanical disruption with ceramic beads using a FastPrep-24 5G tissue homogenizer (MP Biomedicals, Inc., Irvine, CA, USA). Plasma and milk samples were used as taken. Ten µL of each sample was analyzed as follows: Samples were mixed with isotopically labeled internal standards and dried under nitrogen flow (Nitrogen evaporator VLM GmbH, Bielefeld, Germany). Afterward, metabolites were derivatized with 5% phenyl isothiocyanate (PITC) (Sigma-Aldrich Chemie GmbH, Taufkirchen, Germany) and extracted with 5 mM ammonium acetate (Sigma-Aldrich Chemie GmbH, Taufkirchen, Germany in methanol (Roth, Karlsruhe, Germany). One aliquot of the extract was used for liquid chromatography-mass spectrometry (LC-MS) analysis of biogenic amines and amino acids. A second aliquot was used for flow injection analysis-tandem mass spectrometry (FIA-MS/MS) to analyze lipids and hexoses. Both types of measurements were performed in-house (core facility, University of Hohenheim) on a QTRAP mass spectrometer applying electrospray ionization (ESI) (AB Sciex API 5500Q-TRAP, AB Sciex Germany GmbH (sciex.com), Darmstadt, Germany). The metabolite profiling of the liver supernatants, plasma and milk samples was carried out by using the AbsoluteIDQ p180 Kit (Biocrates Life Science AG, Innsbruck, Austria; https://biocrates.com/absoluteidq-p180-kit/) (accessed on 17 April 2021). This kit identifies and quantifies up to 188 metabolites from 5 compound classes: acylcarnitines (40), proteinogenic and modified amino acids (19), glycerophospho- and sphingolipids (76 phosphatidylcholines, 14 lysophosphatidylcholines and 15 sphingomyelins), biogenic amines (19) and hexoses (1). All reagents used in the processing and analysis were of LC-MS grade. The processed concentration data obtained from the LC-MS analysis were first log-transformed, then centered and Pareto-scaled (MetaboAnalyst 5.0, https://www.metaboanalyst.ca/home.xhtml accessed on 2 October 2021) [30]. 

Due to the low number of animals per group, any complex mathematical modeling had low validity. However, to visualize the total variation between the treatment groups, partial least square-discriminant analyses (PLS-DA) were performed for each metabolite profile in the liver, plasma and milk, followed by determining cross-validation metrics Q2. Furthermore, data were analyzed by hierarchical Euclidean clustering, and the top 50 metabolites were visualized in heatmaps, also to demonstrate interindividual variation. PLS-DA, clustering and heatmaps were generated by using MetaboAnalyst 5.0 [30]. Statistical relevance of group differences for top 50 metabolites of interest (absolute metabolite concentrations in µmol/L; selected by cluster analysis) was assessed by using One-Way ANOVA with Dunnett’s multiple comparison post-test (comparing control group M with each of the treatment groups PA 1, 2 and 3) using GraphPad.Prism 9.3.0 (https://www.graphpad.com) (accessed on 1 March 2022). Significance level was set at *p* < 0.05.

## Figures and Tables

**Table 1:** Liver metabolite profiles affected by PA treatment as selected by Euclidean clustering. Differences were tested by using One-Way ANOVA and by using Dunnett’s multiple comparison post-test; significances were determined between molasses control (M) and each pyrrolizidine treatment group (PA1–3).

Metabolite/Treatment ^1^	M	PA1	PA2	PA3	*p* ^2^
C3-DC (C4-OH)	0.029 ± 0.002 ^a^	0.028 ± 0.002	0.030 ± 0.003	0.041 ± 0.003 ^b^	0.018
C18:2	0.024 ± 0.001 ^c^	0.027 ± 0.001	0.030 ± 0.001	0.035 ± 0.004 ^d^	0.020
C5:1	0.088 ± 0.003 ^a^	0.089 ± 0.006	0.097 ± 0.007	0.110 ± 0.007 ^b^	0.037
C14:1-OH	0.037 ± 0.004 ^a^	0.034 ± 0.001	0.044 ± 0.005	0.058 ± 0.008 ^b^	0.029
C16:2-OH	0.038 ± 0.002	0.036 ± 0.001	0.045 ± 0.004	0.053 ± 0.007	n.s.
C16:1	0.032 ± 0.002 ^c^	0.035 ± 0.002	0.041 ± 0.002	0.047 ± 0.005 ^d^	0.014
C18:1-OH	0.065 ± 0.009	0.071 ± 0.004	0.082 ± 0.006	0.092 ± 0.007	n.s.
C14:2-OH	0.080 ± 0.004 ^a^	0.087 ± 0.001	0.086 ± 0.002	0.100 ± 0.009 ^b^	0.028
C7-DC	0.058 ± 0.005	0.053 ± 0.005	0.070 ± 0.007	0.085 ± 0.011	n.s.
C16-OH	0.029 ± 0.001 ^c^	0.028 ± 0.001	0.037 ± 0.004	0.049 ± 0.006 ^d^	0.009
C14:2	0.022 ± 0.003 ^a^	0.021 ± 0.002	0.029 ± 0.004	0.036 ± 0.005 ^b^	0.042
C14:1	0.039 ± 0.003 ^c^	0.037 ± 0.003	0.045 ± 0.004	0.071 ± 0.008 ^d^	0.001
C12:1	0.071 ± 0.005 ^a^	0.066 ± 0.003	0.072 ± 0.002	0.091 ± 0.007 ^b^	0.010
C9	0.025 ± 0.002 ^e^	0.043 ± 0.005	0.075 ± 0.007 ^f^	0.081 ± 0.011 ^f^	0.0004
C18	0.091 ± 0.016 ^a^	0.084 ± 0.007	0.140 ± 0.012 ^b^	0.100 ± 0.004	0.017
C8	0.120 ± 0.007	0.110 ± 0.008	0.120 ± 0.005	0.140 ± 0.097	0.025
C5-M-DC	0.070 ± 0.005	0.064 ± 0.005	0.085 ± 0.003	0.088 ± 0.009	n.s.
C18:1	0.046 ± 0.006	0.043 ± 0.003	0.058 ± 0.006	0.059 ± 0.002	n.s.
C6:1	0.140 ± 0.005 ^a^	0.130 ± 0.005	0.110 ± 0.024	0.063 ± 0.023 ^b^	0.035
C3:1	0.049 ± 0.004	0.045 ± 0.005	0.027 ± 0.008	0.025 ± 0.009	n.s.
PC ae C32:1 ^3^	3.400 ± 0.300	3.600 ± 0.200	3.800 ± 0.200	2.500 ± 0.300	0.020
SM C20:2 ^3^	0.390 ± 0.020	0.420 ± 0.060	0.450 ± 0.020	0.320 ± 0.010	n.s.
putrescine	1.3 ± 0.150 ^a^	0.640 ± 0.090 ^b^	1.10 ± 0.180	0.960 ± 0.130	0.028
phenylethylamine	0.19 ± 0.090	0.010 ± 0.002	0.04 ± 0.030	0.440 ± 0.390	n.s.

Concentrations of metabolites (µmol/L) are given as means ± SEM, *n* = 4 animals/group; ^1^ M = Control, PA1 = 0.47 mg PA/kg body weight/day, PA2 = 0.95 mg PA/kg body weight/day and PA3 = 1.91 mg/kg body weight/day; ^2^ *p* = probability of treatment effects (*p* values of One-Way ANOVA analysis). ^3^ These metabolites of PC and SM substrate classes were analyzed exemplarily. Further metabolites of these classes are demonstrated in Figure 1. ^a,b; c,d; e,f^ Means with different superscripts within a row indicate significant differences between group M and PA1, 2 and 3 (^a,b^ *p* ≤ 0.05; ^c,d^ *p* ≤ 0.01; ^e,f^ *p* ≤ 0.001), n.s. not significant. Metabolite abbreviations C3-DC (C4-OH) malonylcarnitine (hydroxybuturylcarnitine), C18:2 octadecadienylcarnitine, C5:1 tiglylcarnitine, C14:1-OH hydroxytetradecenoylcarnitine, C16:2-OH hydroxyhexadecadienoyl, C16:1 hexadecenoylcarnitine, C18:1-OH hydroxyoctadecenoylcarnitine, C14:2-OH hydroxytetradecadienoylcarnitine, C7-DC pimeloylcarnitine, C16-OH hydroxyhexadecanoylcarnitine, C14:2 tetradecadienoylcarnitine, C14:1 tetradecenoylcarnitine, C12:1 dodecenoylcarnitine, C9 nonaylcarnitine, C18 Octadecanoylcarnitine, C8 Octanoylcarnitine, C5-M-DC methylglutarylcarnitine, C18:1 octadecenoylcarnitine, C6:1 hexenoylcarnitine, C3:1 propenoylcarnitine, PC ae C32:1 phosphatidylcholine C32:1 and SM C20:2 sphingomyelin C20:2.

**Table 2:** Plasma metabolite profiles affected by PA treatment as selected by Euclidean clustering. Differences were tested by using One-Way ANOVA and by using Dunnett’s multiple comparison post-test; significances were determined between molasses control (M) and each pyrrolizidine treatment group (PA1–3).

Metabolite/Treatment ^1^	M	PA1	PA2	PA3	*p* ^2^
C16:2-OH	0.028 ± 0.002 ^a^	0.033 ± 0.003	0.034 ± 0.002	0.036 ± 0.002 ^b^	0.090
C14:1-OH	0.025 ± 0.001	0.026 ± 0.002	0.034 ± 0.003	0.036 ± 0.005	0.060
C3-DC (C4-OH)	0.036 ± 0.002	0.038 ± 0.003	0.048 ± 0.006	0.049 ± 0.003	0.090
C6 (C4:1-DC)	0.191 ± 0.002	0.202 ± 0.010	0.233 ± 0.019	0.225 ± 0.005	0.070
C14:1	0.031 ± 0.002 ^a^	0.033 ± 0.001	0.042 ± 0.005	0.045 ± 0.004 ^b^	0.030
C16-OH	0.031 ± 0.002	0.031 ± 0.002	0.038 ± 0.004	0.037 ± 0.002	n.s.
C5:1	0.137 ± 0.007	0.139 ± 0.004	0.158 ± 0.011	0.153 ± 0.004	n.s.
C10	0.137 ± 0.004	0.143 ± 0.006	0.159 ± 0.012	0.166 ± 0.010	n.s.
C10:2	0.086 ± 0.004	0.083 ± 0.002	0.086 ± 0.004	0.098 ± 0.006	0.090
Phe	49.60 ± 2.390	53.35 ± 3.260	64.10 ± 4.970	37.08 ± 4.670	0.004
Met	22.78 ± 1.980	23.70 ± 2.260	26.55 ± 2.380	15.50 ± 1.790	0.018
Tyr	37.90 ± 4.830	43.13 ± 4.960	50.35 ± 5.060	30.50 ± 4.980	0.080
Ile	135.8 ± 5.060	143.3 ± 14.34	184.5 ± 21.45	109.9 ± 11.94	0.023
Arg	73.18 ± 4.950 ^a^	65.33 ± 9.490	85.18 ± 7.810	45.65 ± 5.110 ^b^	0.013
Leu	141.3 ± 8.240 ^a^	131.3 ± 19.21	158.8 ± 10.26	93.50 ± 9.450 ^b^	0.020
Val	254.8 ± 15.80	266.0 ± 23.30	277.5 ± 14.90	188.0 ± 18.50	0.021
His	56.08 ± 8.270 ^a^	34.43 ± 15.07	28.10 ± 7.400	19.50 ± 2.070 ^b^	0.090
Cit	102.9 ± 10.30 ^a^	78.88 ± 7.570	94.20 ± 3.090	76.18 ± 5.010 ^b^	0.060
PC aa C42:1 ^3^	0.237 ± 0.010	0.169 ± 0.030	0.169 ± 0.024	0.167 ± 0.010	0.070
PC ae C40:3 ^3^	1.180 ± 0.100 ^a^	1.230 ± 0.080	1.120 ± 0.040	0.890 ± 0.060 ^b^	0.030
SM C16:1 ^3^	15.43 ± 0.650	14.70 ± 1.080	16.15 ± 1.130	13.48 ± 0.780	n.s.

Concentrations of metabolites (µmol/L) are given as means ± SEM, *n* = 4 animals/group; ^1^ M = Control, PA1 = 0.47 mg PA/kg body weight/day, PA2 = 0.95 mg PA/kg body weight/day and PA3 = 1.91 mg/kg body weight/day; ^2^ *p* = probability of treatment effects (*p* values of One-Way ANOVA analysis). ^3^ These metabolites of PC and SM substrate classes were analyzed exemplarily. Further metabolites of these classes are demonstrated in Figure 2. ^a,b^ Means with different superscripts within a row indicate a significant difference (^a,b^ *p* ≤ 0.05), n.s. not significant. Metabolite abbreviations C16:2-OH hydroxyhexadecadienoyl, C14:1-OH hydroxytetradecenoylcarnitine, C3-DC (C4-OH) malonylcarnitine (hydroxybuturylcarnitine), C6 (C4:1-DC) hexanoylcarnitine (fumarylcarnitine), C14:1 tetradecenoylcarnitine, C16-OH hydroxyhexadecanoylcarnitine, C5:1 tiglylcarnitine, C10 decanoylcarnitine, C10:2 decadienylcarnitine, PC aa C42 phosphatidylcholine diacyl C42:1, PC ae C40:3 phosphatidylcholine acyl-alkyl C40:3 and SM C16:1 sphingomyelin C16:1.

**Table 3:** Milk metabolite profiles affected by PA treatment as selected by Euclidean clustering. Differences were tested by using One-Way ANOVA and by using Dunnett’s multiple comparison post-test; significances were determined between molasses control (M) and each pyrrolizidine treatment group (PA1–3).

Metabolite/Treatment ^1^	M	PA1	PA2	PA3	*p* ^2^
C10:1	0.058 ± 0.002 ^a^	0.065 ± 0.003	0.064 ± 0.002	0.080 ± 0.008 ^b^	0.030
C14:1	0.035 ± 0.005	0.034 ± 0.001	0.045 ± 0.006	0.058 ± 0.010	0.080
C14:2-OH	0.218 ± 0.018 ^a^	0.228 ± 0.007	0.222 ± 0.016	0.303 ± 0.025 ^b^	0.020
C16:2-OH	0.064 ± 0.007	0.073 ± 0.009	0.061 ± 0.007	0.086 ± 0.008	n.s.
C14	3.480 ± 0.220 ^c^	3.207 ± 0.140	3.980 ± 0.320	4.910 ± 0.280 ^d^	0.002
C10	0.138 ± 0.006 ^a^	0.143 ± 0.011	0.155 ± 0.008	0.190 ± 0.017 ^b^	0.030
C18:1-OH	0.357 ± 0.013 ^c^	0.416 ± 0.031	0.507 ± 0.024	0.611 ± 0.082 ^d^	0.010
C18:1	0.066 ± 0.001 ^a^	0.077 ± 0.004	0.081 ± 0.006	0.094 ± 0.010 ^b^	0.050
C14:2	0.024 ± 0.003	0.022 ± 0.002	0.023 ± 0.001	0.022 ± 0.004	n.s.
C18	0.061 ± 0.005	0.066 ± 0.009	0.075 ± 0.008	0.093 ± 0.013	n.s.
C16:2 ^3^					n. appl.
C12-DC	0.213 ± 0.004	0.227 ± 0.010	0.212 ± 0.008	0.234 ± 0.006	n.s.
C12	3.660 ± 0.190 ^c^	4.230 ± 0.260	4.060 ± 0.150	4.740 ± 0.190 ^d^	0.020
C12:1	2.320 ± 0.100 ^a^	2.680 ± 0.170	2.570 ± 0.100	2.920 ± 0.130 ^b^	0.040
C10:2	0.085 ± 0.008 ^a^	0.099 ± 0.001	0.079 ± 0.006	0.106 ± 0.003 ^b^	0.020
C6:1	0.100 ± 0.024 ^a,c^	0.132 ± 0.006	0.032 ± 0.017 ^d^	0.040 ± 0.003 ^b^	0.0003
PC aa C36:0 ^4^	0.943 ± 0.051 ^a^	1.223 ± 0.048 ^b^	1.090 ± 0.058	1.240 ± 0.091 ^b^	0.020
SM C26:0 ^4^	0.051 ± 0.003 ^c^	0.064 ± 0.003	0.056 ± 0.005	0.072 ± 0.004 ^d^	0.009
kynurenine	0.082 ± 0.008 ^a^	0.185 ± 0.044 ^b^	0.151 ± 0.027	0.102 ± 0.013	0.070
His	4.900 ± 0.810 ^c^	1.920 ± 0.540 ^d^	1.700 ± 0.530 ^d^	1.320 ± 0.350 ^d^	0.003
spermidine	0.480 ± 0.050	0.760 ± 0.140	0.400 ± 0.050	0.420 ± 0.030	0.030
spermine	1.600 ± 0.210	1.890 ± 0.240	0.870 ± 0.190	1.550 ± 0.260	0.040

Concentrations of metabolites (µmol/L) are given as means ± SEM, *n* = 4 animals/group; ^1^ M = Control, PA1 = 0.47 mg PA/kg body weight/day, PA2 = 0.95 mg PA/kg body weight/day and PA3 = 1.91 mg/kg body weight/day; ^2^ *p* = probability of treatment effects (*p* values of One-Way ANOVA analysis). ^3^ C16:2 has missing values in PA2 group, thus statistical analysis is not possible (not applicable, n.appl.). ^4^ These metabolites of PC and SM substrate classes were analyzed exemplarily. Further metabolites of these classes are demonstrated in Figure 3. ^a,b; c,d^ Means with different superscripts within a row indicate a significant difference (^a,b^ *p* ≤ 0.05; ^c,d^ *p* ≤ 0.01), n.s. not significant. Metabolite abbreviations C10:1 decenoylcarnitine, C14:1 tetradecenoylcarnitine, C14:2-OH hydroxytetradecanienylcarnitine, C16:2-OH hydroxyhexadecadienoyl, C14 tetradecanoylcarnitine, C10 decanoylcarnitine, C18:1-OH hydroxyoctadecenoylcarnitine, C18:1 octadecenoylcarnitine, C14:2 tetradecadienoylcarnitine, C18 octadecanoylcarnitine, C16:2 hexadecadienylcarnitine, C12-DC dodecenoylcarnitine, C12 dodecanoylcarnitine, C12:1 dodecenoylcarnitine, C10:2 decadienylcarnitine, C6:1 hexenoylcarnitine, PC aa C36:0 phosphatidylcholine diacyl C36:0 and SM C26:0 sphingomyelin C26:0.

## Data Availability

The data presented in this study are available on request from the corresponding author.

## Supplementary Materials

The following supporting information can be downloaded at: https://www.mdpi.com/article/10.3390/toxins15100601/s1, Figure S1: Partial least square discriminant analysis (PLS-DA, (A) and cross-validation metrics Q2 (B)) of liver metabolite profile in dairy cows treated with different concentrations of pyrrolizidine alkaloids (M = Control, PA1 = 0.47 mg PA/kg body weight/day, PA2 = 0.95 mg PA/kg body weight/day, PA3 = 1.91 mg/kg body weight/day). Figure S2: Partial least square discriminant analysis (PLS-DA, (A) and cross validation metrics Q2 (B)) of plasma metabolite profile in dairy cows treated with different concentrations of pyrrolizidine alkaloids (M = Control, PA1 = 0.47 mg PA/kg body weight/day, PA2 = 0.95 mg PA/kg body weight/day, PA3 = 1.91 mg/kg body weight/day). Figure S3: Partial least square discriminant analysis (PLS-DA, (A) and cross validation metrics Q2 (B)) of milk metabolite profile in dairy cows treated with different concentrations of pyrrolizidine alkaloids (M = Control, PA1 = 0.47 mg PA/kg body weight/day, PA2 = 0.95 mg PA/kg body weight/day, PA3 = 1.91 mg/kg body weight/day).
