# Supplementary material for: Metabolite Profiling in the Liver, Plasma and Milk of Dairy Cows Exposed to Tansy Ragwort (Senecio jacobae) Pyrrolizidine Alkaloids
Source: Toxins (Basel). 2023 Oct 6;15(10):601. doi: 10.3390/toxins15100601 (PMC10610733; doi:10.3390/toxins15100601)
Supplement: Supplementary file 1 [file toxins-15-00601-s001.zip › toxins-2560439-supplementary.pdf]

toxins

MDPI

Supplementary Materials: Metabolite Profiling in the Liver, Plasma and Milk of Dairy Cows Exposed to Tansy Ragwort (*Senecio jacobae*) Pyrrolizidine Alkaloids

A

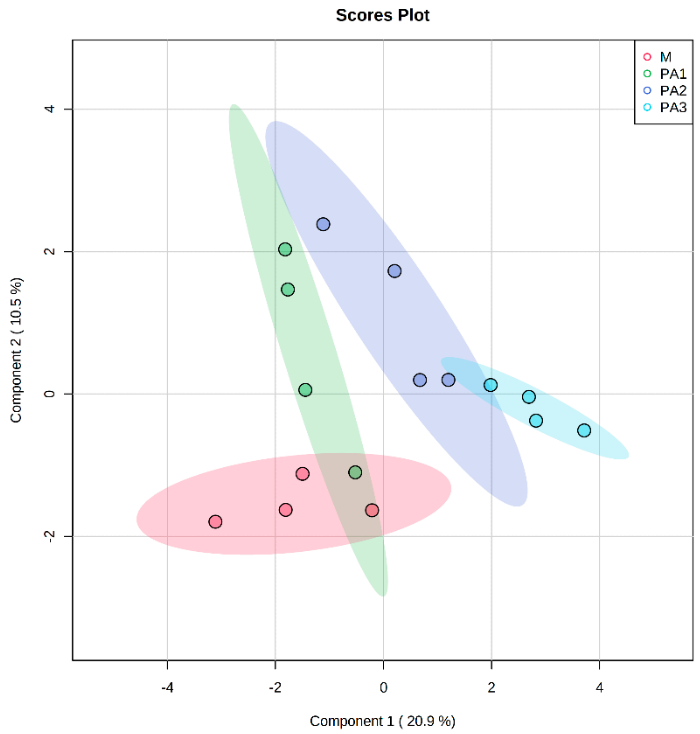

B

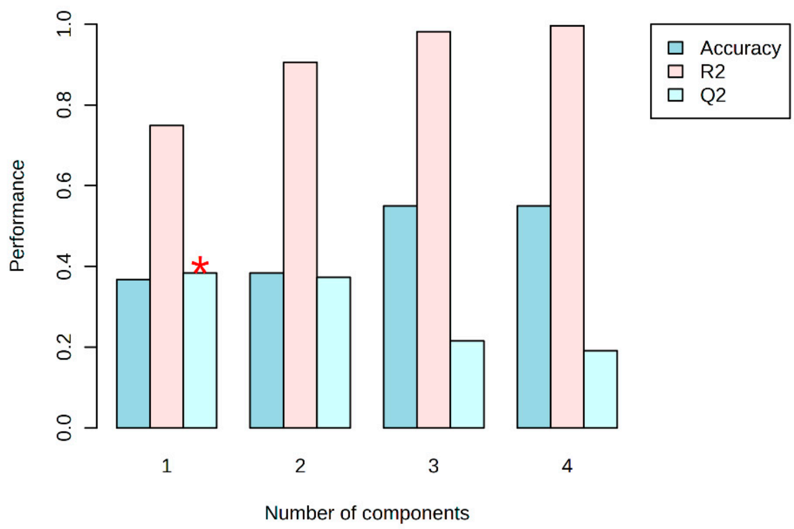

Figure S1 Partial least square discriminant analysis (PLS-DA, (A) and cross-validation metrics Q2 (B)) of **liver** metabolite profile in dairy cows treated with different concentrations of pyrrolizidine alkaloids (M = Control, PA1 = 0.47 mg PA/kg body weight/day, PA2 = 0.95 mg PA/kg body weight/day, PA3 = 1.91 mg/kg body weight/day).

A

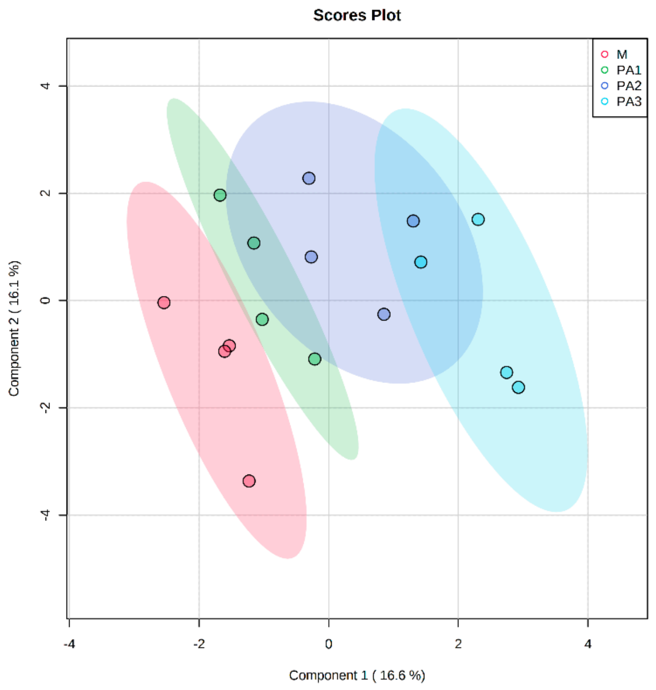

B

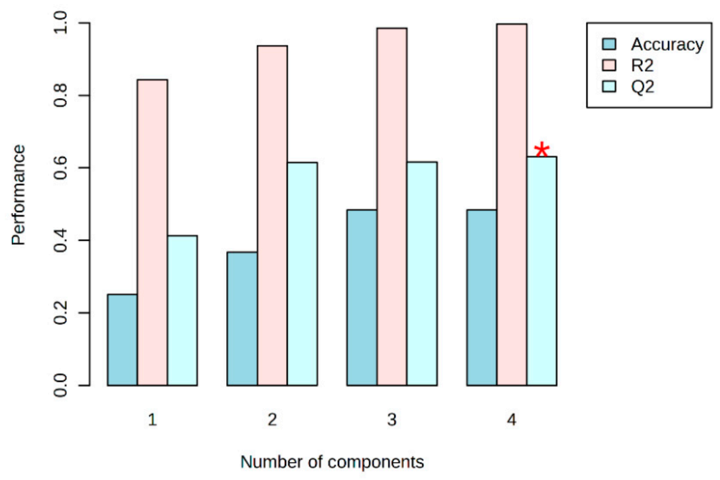

Figure S2 Partial least square discriminant analysis (PLS-DA, (A) and cross validation metrics Q2 (B)) of **plasma** metabolite profile in dairy cows treated with different concentrations of pyrrolizidine alkaloids (M = Control, PA1 = 0.47 mg PA/kg body weight/day, PA2 = 0.95 mg PA/kg body weight/day, PA3 = 1.91 mg/kg body weight/day).

A

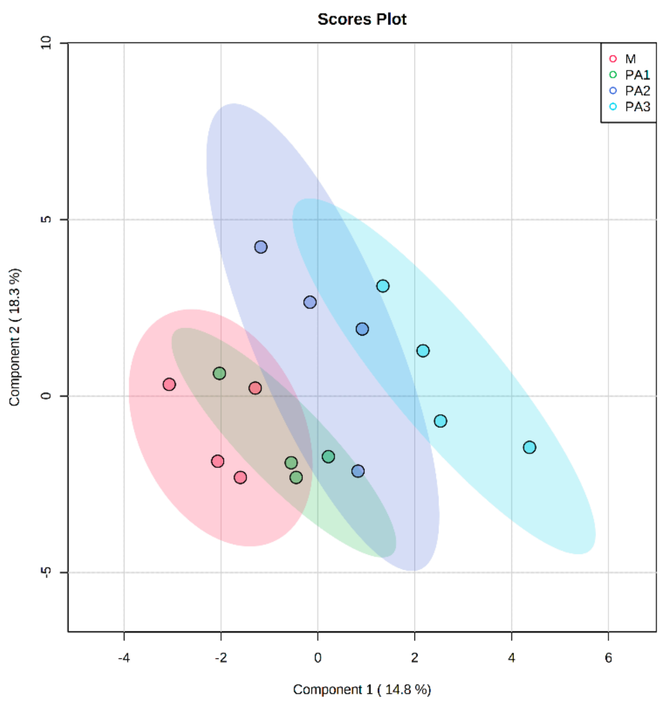

B

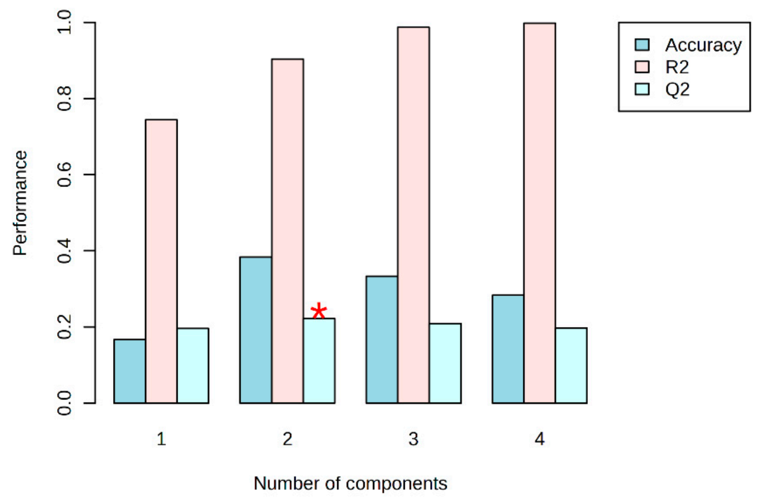

Figure S3 Partial least square discriminant analysis (PLS-DA, (A) and cross validation metrics Q2 (B)) of **milk** metabolite profile in dairy cows treated with different concentrations of pyrrolizidine alkaloids (M = Control, PA1 = 0.47 mg PA/kg body weight/day, PA2 = 0.95 mg PA/kg body weight/day, PA3 = 1.91 mg/kg body weight/day).
